# INTERCONNECTIONS BETWEEN COGNITION, SKELETAL MUSCLES, AND PHYSICAL FUNCTION IN ETHNICALLY DIVERSE COHORTS

**DOI:** 10.1093/geroni/igae098.0790

**Published:** 2024-12-31

**Authors:** Caterina Rosano, Stephen Kritchevsky, Paul Coen

**Affiliations:** Chair; Co-Chair; Discussant

## Abstract

There is marked variability in mobility function at older ages, yet some maintain high functioning even in the face of adverse locomotor risk factors. The mechanisms underlying this resilience remain elusive, but it is clear that the central nervous system plays an important role. Recent findings have shed light on the interconnected biological processes involving muscle-brain cross-talk, albeit primarily in animal models and specific patient populations with muscle degeneration. The extent and nature of such cross-talk in older adult populations remain debated. This symposium aims to advance our understanding of brain-muscle interactions by delving into this emerging concept. Comprising four talks, the symposium will explore various facets of muscle health across diverse cohorts of older adults in the United States. Topics include the structure of the associations between measures of cognitive and physical functions in 3 studies (SOMMA, B-NET and Dunedin), the role of muscle mitochondrial function in the SOMMA study, and the role of intermuscular adiposity in skeletal muscles in the MESA and CARDIA studies. These observations suggest distinctive modulatory and adaptive capacities of the central nervous and musculoskeletal systems may offer insights into understanding the origins of mobility resilience by facilitating adaptation and compensation. This conceptual framework holds potential for identifying innovative multi-systemic interventions aimed at enhancing mobility resilience in older adults.

